# Are There Modifiable Environmental Factors Related to Amyotrophic Lateral Sclerosis?

**DOI:** 10.3389/fneur.2018.00220

**Published:** 2018-04-06

**Authors:** Bozenna Kuraszkiewicz, Teresa Podsiadły-Marczykowska, Hanna Goszczyńska, Maria Piotrkiewicz

**Affiliations:** Department of Brain Imaging and Investigation of Nervous System Function, Nalecz Institute of Biocybernetics and Biomedical Engineering, Polish Academy of Sciences, Warsaw, Poland

**Keywords:** amyotrophic lateral sclerosis, risk factors, trial size, age factor, dietary habits, gut microbiome, psychological stress, oxidative damage

Amyotrophic lateral sclerosis (ALS) is a fatal adult-onset neurodegenerative disorder of unknown etiology and no preventive treatment. It is characterized by degeneration of both upper motoneurons in the primary motor cortex and lower motoneurons in the brainstem and spinal cord. It results in progressive muscular weakness and atrophy, leading eventually to respiratory insufficiency and death. Approximately 50% of patients with ALS die within 30 months of symptom onset (1). Although ALS is considered a rare disease (incidence between 0.6 and 2.6 new cases per year per 100,000, prevalence 3–8 per 100,000), it is in fact more common than generally recognized, with an adult lifetime risk of 1 in 400 (2). Approximately 10% of all ALS cases are familial (fALS), and the remaining 90% are sporadic (sALS). Genetic factors are involved in the pathogenesis of fALS and up to 15% of sALS (3, 4).

Nowadays, the only established risk factors for ALS are advanced age, male gender, and certain genetic mutations (1). Given that no more than 25% of all ALS diagnosis has genetic basis, for the remaining percentage of cases gene–environmental interaction may be responsible.

In the recent years, a vast number of studies addressing ALS risks related to the exposure to environmental factors have been published [for a recent review, see Ref. (5)]. Their analysis is difficult, since they applied various methods of data collection and analysis, from studying of toxicants’ content in tissue samples, through case–control studies and analysis of death certificates, to reviews and meta-analyses. Original studies were usually performed on small local patient cohorts, and their results are ambiguous and often contradictory. Virtually all so far published reviews on ALS risk factors were inconclusive and called for further investigations.

Nevertheless, as mentioned earlier, at least 75% of ALS cases do not have established genetic basis, so the search for modifiable environmental factors continues. The research in this field is important, since it may lead to formulation of measures, which could prevent this fatal disease or at least delay its development. Therefore, we decided to put forward some thoughts that arose from studying published literature, hoping that they could help to design future successful studies on ALS risk factors.

## The Size of an Individual Trial

Systematic reviews and meta-analyses are often viewed as tools which may overcome weaknesses of small trials. However, as stated by Bartolucci and Hillegass (6), “… one should keep in mind that meta-analyses should neither be a replacement for well-designed large-scale randomized studies nor a justification for conducting small underpowered studies.” Moreover, it has been pointed out that individual small trials are more prone to biases, which will affect final conclusions of meta-analyses (7). That is actually one of the main drawbacks of so far reported research on ALS risk factors. Even in the most exhaustive review recently published (5), the meta-analyses were based on many studies with small sample size (see Table 1). Evidently, the authors were not able to find sufficient number of papers with acceptable homogeneity and eventually concluded: “…as the number of studies included in most meta-analyses in this study was small, caution should be used when interpreting the findings.” Summing up, if future meta-analyses are to produce reliable results, they should be based on the properly designed original studies of sufficiently large size. The description of methods to calculate a sufficient size of the study, which aims toward determination of the effect of a given factor on the ALS risk, can be found in Ref. (8). The author provided an example of such calculation for a study aiming at the detection of an informational odds ratio of at least 2.0 at the α = 0.05 level of statistical significance when the proportion of non-diseased individuals, who are exposed to a given environmental risk, equals 0.10. In such a case, to obtain a sufficient power (>80%), the study needs 200 diseased and 200 non-diseased participants. As can be seen in Table 1, this number of patients was exceeded only in few studies included in meta-analyses of Wang et al.

**Table 1:** Exposure to environmental factors: results of meta-analyses (5).

Risk factor	Papers No.	Publication dates	Patients			Controls			Significance *P*	OR (95% CI)
			No.	Range	*n*^a^	No.	Range	*n*^a^		
Heavy metals (lead)	11 (7)	1981–2009	1,459	56–518	1	1,887	66–518	3	<0.00001	1.71 (1.38, 2.11)
Agricultural chemicals (pesticides)	12 (10)	1993–2013	2,144	41–661	2	99,612	64–84,698	6	0.0001	1.57 (1.43–6.3)
Organic solvents	7	1991–2010	701	36–174	0	1,286	92–348	3	0.007	1.43 (1.10–1.86)
Previous trauma	17	1981–2012	2,955	25–518	5	3,763	50–518	6	<0.00001	1.73 (1.43–2.09)
Electric shock	6	1983–1999	961	25–518	1	1,154	50–518	2	<0.0001	3.27 (1.87–5.83)
Professional sports	4	1998–2012	824	61–377	2	1,082	112–377	3	Significant	1.35 (1.11–1.65)

*^a^Number of studies with >200 participants*.

As ALS is a rare disease, large studies can be performed only in international cooperation. An effort to create the big ALS database was undertaken in the ongoing project “OnWebDUALS,” aiming toward collection and subsequent analysis of standardized questionnaires from several hundreds of patients (http://www.neurodegenerationresearch.eu/wp-content/uploads/2015/02/ONWebDUALS.pdf). The statistical analysis applying modern methodology will take into account patients’ genetic and phenotypic diversity and hopefully will come with novel associations between individual factors and ALS risk. USA National ALS Registry (9) and Australian Web database (10) should also be mentioned. The need for big data was acknowledged by European Commission who recently issued a call *SC1-DTH-08-2018: Prototyping a European interoperable Electronic Health Record exchange*, aiming to merge together individual patient databases existing in different European countries for increasing their statistical power. It is worth trying to use big data to conduct studies on those risk factors, which have been shown to relate to known mechanisms responsible for ALS development, in particular factors collected in Table 1.

## Age Factor

The incidence of ALS increases with age and peaks in mid seventies, with very few new cases below 25 and above 90 years old (11, 12). There is an “age-related cascade of neurodegeneration” (13), whereby accumulative oxidative damage provokes metabolic deficiencies, protein aggregation, decline in mitochondrial function, and inflammation (14–16). There is also evidence of heavy metals accumulation with age in spinal interneurons and in motoneurons (17). Exposure to these and other environmental pathogens will accelerate neurodegeneration cascade, which will be accompanied by telomere shortening, a widely known determinant of cell senescence. Indeed, accelerated telomere shortening was observed in ALS mice (18) and in leukocytes from sporadic ALS patients (19). Telomere shortening in ALS certainly deserves more attention, since it may be modified by a healthy lifestyle (20). Summing up, susceptibility to the environmental risks would increase with age that is in line with abovementioned age dependency of ALS incidence. The future studies might be presumably more conclusive if the analysis was carried on in distinct age groups.

## Dietary Habits

The studies on possible influences of diet on ALS are rather scarce. A higher ALS risk was associated with increased dietary uptake of fat and glutamate (21, 22). A decreased risk was observed in individuals whose diet was rich in fiber (21) or green–yellow vegetables (23). Higher intake of antioxidants and carotenes from fruit and vegetables was associated with better patient’s condition [higher ALSFRS-R scores (24)]. In several studies, a long-term vitamin E supplementation was associated with lower ALS rates (25, 26), although in some other studies such a relationship was not observed [e.g., Ref. (21, 27)]. An interesting observation was made by Felmus et al. (28, 29). They found that ALS patients drank more milk than control subjects. To the best of our knowledge, milk was not considered a potential ALS risk factor in any other study. However, diary consumption was shown to be associated with increased risk of Parkinson’s disease (30). Summing up, we think that more attention should be paid to the effects of diet, especially in context of its antioxidant capacity. Diary consumption should also be taken into account as the potential risk factor.

## Gut Microbiome

The interest in the role of gut microbiome in health and disease is recently growing (31, 32). The importance of healthy gut microbiome has been shown in three most recent articles published in Science, reporting that the patients with low levels of the bacterium *Akkermansia muciniphila* poorly responded to antitumor treatment (33–35). Moreover, a few papers have recently highlighted a potential link of gut dysbiosis and neurodegenerative diseases (35–38). Fang et al. (39) found significantly increased population of harmful microorganisms (genus *Dorea*) and significantly reduced population of beneficial microorganisms (genus *Oscillibacter, Anaerostipes, Lachnospiraceae*) in ALS patients and suggested that the imbalance in intestinal microflora constitution has a strong association with ALS pathogenesis. Recent studies revealed signs of leaky intestine in G93A ALS mouse model (40, 41), which were associated with reduced levels of *Butyrivibrio fibrisolvens, Escherichia coli*, and *Fermicus*, as compared with wild-type mice. The leaking intestine might be at least partially responsible for the observed discordance between increased presymptomatic total daily energy intake and decreased presymptomatic body mass index in ALS patients (22). These aspects are worth considering in future studies.

## Psychological Stress

The studies investigating association of ALS with psychological stress are very rare. The study of Okamoto et al. (23), who observed increased ALS risk in patients reporting “type A behavior” and high or moderate level of stress, was the only one directly addressing this issue, which we were able to find. Type A behavior was not defined in this study and the self-reported stress intensity was based on 4-point scale (never/hardly, light, moderate, and high). Nevertheless, psychological stress is significantly associated with higher oxidative stress, lower telomerase activity, and shorter telomere length (42), all involved in ALS. It should also be noted that the most severe telomere shortening was found to be associated with stress occurring in childhood (43). Therefore, we think that the retrospective questionnaires should address the stressful events, with special attention paid to those happening in childhood.

## Concluding Remarks

Summing up, we think that future studies on ALS risk factors should be carefully designed and should involve sufficiently large patient cohorts. The international studies should consider potential risks with established relation to the commonly accepted ALS mechanisms, but also those less investigated but potentially important as gut health, dietary habits, and psychological stress. The evaluation of the last two factors should involve a consensus on the ways to measure their impact.

## Author Contributions

BK, TP-M, and HG: Participation in literature collection, discussion, and contribution to final manuscript. MP: Participation in literature collection, manuscript drafting, discussion, and contribution to final manuscript.

## Conflict of Interest Statement

The authors declare that the research was conducted in the absence of any commercial or financial relationships that could be construed as a potential conflict of interest.
